# Practitioner's Bookshelf

**Published:** 1892-04-23

**Authors:** 


### PRACTITIONERS' BOOKSHELF.

### THE "MEDICAL ANNUAL."*

The "Medical Annual" is one of the most useful books published annually. It is so carefully arranged alphabetically that ready reference is easy, and references to the original articles are so thoroughly given that if it is desired to look them up it can be readily done, and the evidence of the value of any particular method of treatment recommended investigated.

To the writers of articles, the "Medical Annual" is often invaluable, the information being thoroughly up to the date of publication, and references given to the very latest literature on the subjects. Thus we notice one reference to a paper published last November, and another as late as December. It seems quite the object of the writers to have all the information thoroughly up to date.

The writers on the different subjects are men who have specially worked at them. Thus, Mr. Herbert Allingham writes on the rectum, Mr. Hurry Fenwick on the bladder, Mr. Lang on ophthalmic surgery, Dr. Saundby on renal disease, and Dr. Watson Williams on diseases of the throat.

The book begins with a chapter on new drugs, and then Mr. Hurry Fenwick gives a chapter on the electric illumination of the bladder illustrated by a series of very beautiful coloured plates, which show how useful such a method of diagnosis may be. Then the new methods of treatment in each disease are given under the headings of the diseases. This part occupies the greater portion of the book, and is the most valuable. We notice that some of the writers have not confined their attention to treatment, but, like Mr. Hurry Fenwick, have given information with regard to other matters. Thus, we see recently-published cases of acromegaly are described, and recent views on the etiology and pathology of diphtheria are given. Dr. Armand Ruffer also contributes a paper on recent advances in bacteriology, illustrated by several very good coloured plates; and medical photography is dealt with in a way thoroughly helpful to those who wish to start this new line of work. Medical officers of health will find a useful chapter on recent inventions of sanitary appliances, which is particularly well illustrated; and on recent sanitary legislation. A chapter is devoted to new inventions, improvements in pharmacy, dietetic preparations. There is a directory of lunatic asylums, homes for incurables, and hydropathic establishments, and the work ends with a list of the books published during the year on medical and allied subjects.

Pictures of new apparatus and instruments are liberally scattered through the pages, with the addition of several beautiful coloured plates. The book is published at a wonderfully moderate price. We heartily commend it to all medical men.

* The "Medical Annual," 1892. Illustrated. (Bristol: J. Wright and Co. London: Simpkin and Co.)

